# Clustering and Combining Pattern of High-Risk Behaviors among Iranian University Students: A Latent Class Analysis

**Published:** 2017-11-13

**Authors:** Sima Afrashteh, Haleh Ghaem, Abbas Abbasi-Ghahramanloo, Hamid Reza Tabatabaee

**Affiliations:** ^1^ Student Research Committee, Shiraz University of Medical Sciences, Shiraz, Iran; ^2^ Vice-Chancellor for Public Health, Bushehr University of Medical Sciences, Bushehr, Iran; ^3^ Research Center for Health Sciences, Institute of Health, Department of Epidemiology, School of Health, Shiraz University of Medical Sciences, Shiraz, Iran; ^4^ Department of Public Health, School of Health, Ardabil University of Medical Sciences, Ardabil, Iran; ^5^ Department of Epidemiology, Faculty of Health, Iran University of Medical Sciences, Tehran, Iran

**Keywords:** Latent class analysis, High-risk behaviors, Subgrouping, Iran

## Abstract

**Background:** High-risk behaviors are increasing among young adults worldwide. We aimed to identify university students’ subgroups on the basis of high-risk behaviors and to assess the role of age, living alone, religious beliefs, and parental support in the membership of specific subgroups.

**Study design:** A cross-sectional study

**Methods:** The study was conducted in Bushehr (the south of Iran) from November to December 2016. The sample included 977 university students selected through random sampling. The data were collected using a self-administered questionnaire. Then, latent class analysis was used to classify the students.

**Results:** Totally, five latent classes were identified as follows: low risk, high risk, somewhat low risk, hookah user, and very high risk. Notably, 7.7% and 2.5% of the students belonged to high risk and very high risk classes, respectively. The results suggested the protective effect of familial support and religiosity on high-risk behaviors.

**Conclusions:** This study indicated the co-occurrence of high-risk behaviors. The findings can be used to plan and evaluate interventions by considering risk factors and protective factors in universities.

## Introduction

High-risk behaviors are increasing among young adults worldwide ^1^. These problematic risk behaviors have been associated with increased risk of chronic diseases and to adversely influence individuals’ physical as well as mental health contributing to early mortality ^2^. By entering the university, away from families, different new experiences and demands result in lifestyle changes, which subsequently increase the risk of high-risk behaviors among individuals ^3^.

Monitoring The Future (MTF) and European School Survey Project on Alcohol and Other Drugs (ESPAD) are two important studies on high-risk behaviors. MTF investigated the rate of drug abuse from 1975 and ESPAD examined alcohol and drug use among Europeans since 1995. The findings showed a long-term decline in illicit drug and alcohol use until 2015 ^4,5^. Among Asian countries, the prevalence of high-risk behaviors has been high in Thailand, Saudi Arabia, and the Middle East ^6-8^. Relatively high prevalence rates of these behaviors among Iranian students are reported ^1, 9^.

Important factors contributing to high-risk behaviors include low self-esteem, negative peer groups, low socioeconomic status, and poverty. On the other hand, evidence has demonstrated that parental support and monitoring, as well as religious beliefs, significantly decreased the incidence of high-risk behaviors ^3, 10^. Parental monitoring was positively associated with a decline in the use of drugs and alcohol, delayed onset of sexual activities, and reduced delinquent behaviors^11^. Parental support and religion were also two protective factors against cigarette smoking ^12^.

Latent Class Analysis (LCA) was first proposed by Lazarfeld and Henry to identify the optimal number of study classes or latent classes ^13^. The efficacy of LCA in empirically identifying population subgroups or specific high-risk behaviors patterns, such as drug use is reported ^14^.

Given the increasing prevalence of high-risk behaviors among Iranian students ^1, 9^, the present study aimed to identify students subgroups using LCA model based on high-risk behaviors patterns, assess the prevalence of these behaviors, and determine the role of religion and parental support in the membership of individuals in these classes.

## Methods

This cross-sectional study was performed on university students in Bushehr, south of Iran between November and December 2016. The participants were selected through multi-stage sampling. In doing so, in each college (strata), several classes (clusters) were randomly selected and all students in the selected classes were enrolled.

The data were collected using a self-administered questionnaire. All participants completed the questionnaires and were assured about the confidentiality of their information. Informed consent was taken from the participants and the study was approved by Ethics committee of the university. Totally, 977 questionnaires were completed.

### Study tools

At first, a pilot study was conducted on 50 students, which confirmed the reliability of the questionnaire by Cronbach’s alpha = 0.90.

The questionnaire was prepared based on WHO Core Questionnaire and Alcohol, Smoking and Substance Involvement Screening Test (ASSIST) by considering the situation of substance use in Iran. The content of the questionnaire was previously validated by a group of researchers in another study. The validity of the questionnaire was also measured by MPH students of public health ^15^. This questionnaire assessed demographic information and information regarding high-risk behaviors including‏ cigarette smoking, hookah use, alcohol use, illicit drug use (e.g. cannabis, opium, and heroin), extramarital sexual activities, and physical violence.

Kendler’s general religiosity scale, translated into Persian^16^, was used to measure the students’ general religiosity^17^. Some examples of the scale items are as follows: “I ask God for assistance when making big decisions”, “I sense God’s direct and indirect attention to me”, and “I see God’s signs in my life every day”. The scale items were responded through a 5-point scale with the following options: “completely agree, agree, neither agree nor disagree, disagree, and completely disagree”. The Cronbach’s alpha of the scale was 0.97. The minimum and maximum possible values of this questionnaire were 28 and 140 respectively. In this scoring, higher scores indicate higher religious beliefs.

Parental support was measured using Aneshensel and Sucoff’s 13-item questionnaire. Some examples of the scale items are as follows: “My mom or dad makes me trust them” and “They truly understand me”. The scale items were responded through a 5-point scale with the following options: “completely agree, agree, disagree, completely disagree, and no idea”. The Cronbach’s alpha of the scale was 0.86 ^16, 18^. The scores of this test ranged from 13 to 65, with higher scores indicating higher parental support.

### Statistical analysis

LCA was used in data analysis. LCA is a latent categorical variable model, which classifies homogeneous individuals. It assumes that beside the measurement error, the correlation between observed variables could be justified by latent variable categories. By various iterations for the number of identified classes of the latent variable and comparing the frequencies of observed response patterns to expected ones, LCA determines the best model and calculates a statistic similar to χ^2^ called G^2^. Based on G^2^ statistic, Akaike Information Criterion (AIC) and Bayesian Information Criterion (BIC) can be calculated for model selection. For all information criteria, a smaller value represents a more optimal balance of model fit and parsimony. Thus, a model with the minimum AIC or BIC might be selected. In order to perform LCA, six observable variables (i.e., indicators) were used to assess high-risk behaviors as a latent variable. These indicators were cigarette smoking, hookah smoking, alcohol use, illicit drug use, extramarital sexual activities, and physical violence. After finalizing the model, age, religious beliefs, parental support, and living alone were entered into the LCA model as covariates. All analyses were conducted by Proc LCA in SAS 9.2 software (SAS Institute Inc. Cary, NC, USA).

## Results

This study was conducted on 977 students. The mean age of the subjects was 21.11 ± 2.37 years. Among them, 41% were male and 11% were married. The mean and standard deviation of religion and parental support were 112.57‏±20.49 and 50.57‏±10.34, respectively. The prevalence of high-risk behaviors has been presented in Table 1. Accordingly, hookah use in the last year (16.1%), alcohol use in the lifetime (11.9%), and cigarette smoking in the last year (10%) were the most prevalent high-risk behaviors among the students. Moreover, the prevalence of high-risk behaviors was higher among male students.

**Table 1:** Percentages of students responding “Yes” to questions about high-risk behaviors

**Items**	**Male )n=404)**		**Female )n=573)**		**Total (n=977)**	
	**n**	**%**	**n**	**%**	**n**	**%**
Cigarette smoking (last year)	65	16.1	33	5.8	98	10.0
Hookah smoking (last year)	91	22.5	66	11.5	157	16.1
Alcohol use (life time)	81	20.0	35	6.1	116	11.9
Illicit drug use (life time)	60	14.9	26	4.5	86	8.8
Extramarital sexual activities (life time)	69	17.1	26	4.5	95	9.7
Physical violence (last year)	51	12.0	18	3.0	69	7.1

Given the 6 binary variables, a total of 64 response patterns were identified. Different measures of model assessment have been shown in Table 2. Since the degree of freedom of G2 statistic was less than 60 (G2  was distributed approximately as chi-square), the overall significance of the estimated model was computed using G2 statistic. When this index is significant, it means that there is a significant difference between expected and observed frequencies and, subsequently, the fitted model is not appropriate. Hence, models 5, 6, and 7 were not significant. In the next stage, the best-fitted model was selected based on G2 , AIC, and‏ BIC. The model with the lowest G2 , AIC, and‏ BIC values is suitable.

**Table 2:** Comparison of LCA models with different latent classes based on model selection statistics

**No. of Latent** **Class**	**No. of Parameters** **Estimated**	**G** ^2^	**df**	***P*** ** value**	**AIC**	**BIC**	**CAIC**	**Adjusted** **BIC**	**Entropy**	**Maximum** **Log-likelihood**
1	6	616.09	57	0.001	628.09	657.40	663.40	638.34	1.00	-1957.06
2	13	86.00	50	0.001	112.00	175.50	188.50	134.20	0.84	-1692.01
3	20	64.02	43	0.020	104.02	201.71	221.71	138.19	0.80	-1681.02
4	27	51.58	36	0.044	105.58	237.47	264.47	151.71	0.81	-1674.81
5	34	38.48	29	0.112	106.48	272.55	306.55	164.57	0.84	-1668.25
6	41	30.31	22	0.111	112.31	312.57	353.57	182.36	0.90	-1664.17
7	48	24.86	15	0.051	120.86	355.31	403.31	202.86	0.90	-1661.44
8	55	18.59	8	0.017	128.59	397.24	452.24	452.24	0.86	-1658.31
9	62	13.71	1	0.001	137.71	440.55	502.55	243.64	0.84	-1655.87

LCA, latent class analysis; AIC, Akaike information criterion; BIC, Bayesian information criterion

**Figure 1:**
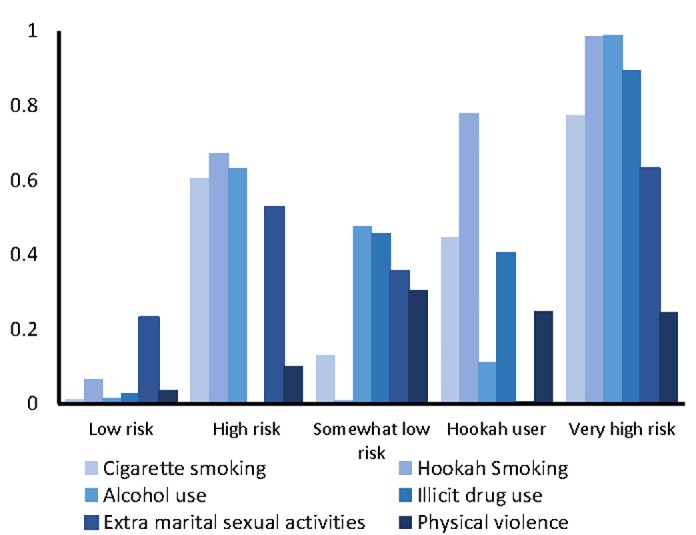
Probability of “Yes” response as a function of class and item in the five-class model of high-risk behaviors

The 5-class model showed the lowest values of G2 , AIC, and‏ BIC. Thus, the 5-class model was selected. The schematic view of item-response probabilities for five-class model presented in Figure 1. After the model was fitted, four main variables (age, religious beliefs, parental support, and living single) were entered into the model as covariates. The frequency of latent classes, the likelihood of endorsing the items, and the odds ratio of covariates associated with latent classes have been presented in Table 3. Accordingly, 79.7% and 2.5% of the students were classified as members of latent class 1 (low risk) and latent class 5 (high risk), respectively.

**Table 3:** The five-class model of high-risk behaviors and its covariates (Latent classes)

**Variables**	**Low-risk**	**High-risk**	**Somewhat** **low-risk**	**Hookah** ** user**	**Very** **high-risk**
Latent class prevalence	0.797	0.077	0.061	0.040	0.025
Item-response probabilities of a ’yes’					
Cigarette smoking (last year)	0.010	0.604	0.130	0.447	0.774
Hookah smoking (last year)	0.065	0.672	0.009	0.779	0.986
Alcohol use (life time)	0.014	0.632	0.476	0.110	0.988
Illicit drug use (life time)	0.026	0.001	0.457	0.406	0.894
Extramarital sexual activities(life time)	0.023	0.526	0.355	0.002	0.631
Physical violence (last year)	0.035	0.099	0.305	0.248	0.246
Covariates (odds ratio)					
Religious beliefs (P<0.001)	Reference	0.209	0.184	0.327	0.368
Parental support (P=0.001)	Reference	0.203	0.648	0.958	0.339
Age (P=0.002)	Reference	1.158	1.220	0.934	0.794
Living alone (P=0.037)	Reference	1.886	2.270	3.010	3.115

**Note:** The probability of a “No” response can be calculated by subtracting the item-response probabilities shown above from 1

Latent class 1, low risk, was characterized by the low probability of high-risk behaviors and latent class 5, very high risk, was characterized by the high probability of all high-risk behaviors, except for physical violence. There were also two other latent classes that reflected different patterns of risk-taking behaviors. Latent class 2, high risk, was characterized by the high probability of cigarette smoking, hookah use, alcohol consumption, and extramarital sexual activities. Latent class 3, somewhat low risk, was characterized by the low probability of high-risk behaviors. However, the probability of high-risk behaviors was higher in the third class than in the first class. Latent class 4, hookah user, was characterized by the high probability of hookah use (77.9%). The odds ratios associated with all covariates have been shown in Table 3. As the table depicts, higher scores of parental support and religious beliefs decreased the odds of membership in the second, third, fourth, and fifth classes in comparison to the first class.

## Discussion

Our findings showed that hookah use (16.1%) was the most prevalent risk behavior among the students. Indeed, male students (22.5%) were two times more likely than females (11.5%) to use hookah. Moreover, the least common high-risk behavior was physical violence (7.1%), which was more prevalent among male students (12%) than in female ones (3%). MTF results also revealed that 32.7% of American students were hookah users in the last year ^4^. The prevalence of hookah use was respectively 17.8% and 11.6% in Tehran and Tabriz college students, which is in accordance with our results ^19, 20^.The increased presence of hookah in public places such as teahouses, more accessibility, affordability, the common use of hookah among family members, lack of sound entertainments for the youth, and tobacco planting in the target community was probably the main causes of high rate of hookah use in this study.

The results of the current study demonstrated that the prevalence of cigarette smoking was 10%. Indeed, this behavior was significantly more prevalent among males (16.1%) compared to females (5.1%). MTF reported the prevalence rate of cigarette smoking to be 22.6% among American college student ^4^. Furthermore, ESPAD in 2015 reported the prevalence rate of lifetime cigarette smoking and smoking over the past thirty days as 46% and 21%, respectively. The highest and lowest prevalence rates of cigarette smoking were observed in the Czech Republic (66%) and Island (16%), respectively ^5^. Among Islamic countries, the prevalence rates of cigarette smoking were 28.1% and 21% and 18.2% in Saudi Arabia, Iraq, and Kuwait, respectively ^21, 23^. Besides, the highest and lowest prevalence rates of cigarette smoking were respectively 39.9% and 13.4% among male students and 25.2% and 0.7% among female students in Iran^24^. In addition, the overall prevalence of cigarette smoking was 9.8% (17.6% in males vs. 4.2% in females), which is in compliance with the present study findings ^25^. However, the prevalence of cigarette smoking in our study was lower compared to other countries, which might be due to the study sample or using different questionnaire..

The present study revealed that the prevalence of alcohol use was 11.9% (20% in males vs. 6.1% in females). The results of MTF showed that the lifetime prevalence of alcohol use was 79.4% in 2014, which was significantly lower compared to 1991 (93.6%) ^4^. The last year prevalence of alcohol use was 6.9% in a study performed in Iran, similar to our study ^26^. Another study reported the lifetime prevalence of alcohol consumption to be 9.6%, which is in compliance with the present study findings ^27^ The lower rate of alcohol use in the present study might be attributed to 1) legal prohibition of alcohol use, 2) disapproval of alcohol use by parents, and 3) cultural and religious stigma against alcohol use.

Our results indicated that lifetime prevalence of illicit drug use was reported to be 8.8% (14.9% in males vs. 4.5% in females). Based on the MTF results, the lifetime prevalence of illicit drug use was 52.4% among college students ^4^. Based on ESPAD (2015), 18% of students used drugs illicitly (21% in males vs. 15% in females) ^5^. Moreover, a study in Zanjan (northwest of Iran) disclosed that 48.2% of male students and 23.4% of female students had used illicit drugs at least once ^28^. Another study found that 57 participants (2.9%) reported lifetime drug use ^26^.

The present study results demonstrated the lifetime prevalence rate of extramarital sexual activities to be 9.7% among college students (17.1% in males and 4.5% in females). According to Youth Risk Behavior Surveillance system, the lifetime and three-month prevalence rates of extramarital sexual activities were 41.2% and 30.1%, respectively ^29^. 10.7% of students in Iran had a history of risky sexual activities, which is in agreement with the findings of the current study ^12^. The lower rate of extramarital sexual activities in our study might be due to the religious and legal prohibition of illegal sexual activities as well as the cultural stigma against such activities in Iran.

In this study, 5 latent classes were identified for high-risk behaviors: low risk (79.7%), high risk (7.7%), somewhat low risk (6.1%), hookah user (4%), and very high risk (2.5%). A previous study evaluated lifestyle and high-risk behaviors among American students and extracted 4 latent classes in female students as follows: 1) poor lifestyle, yet low-risk behaviors (40%), 2) high risk (24.3%)‏, 3)‏ moderate lifestyle, few high-risk behaviors (20.4%), and health conscious (15.4%)‏. Besides, the following four latent classes were extracted in male students: poor lifestyle, low risk (9.2%), high risk (33.6%),‏ moderate lifestyle, low risk (51%), and classic jocks (6.2%) ^30^. Similarly, a study in the U.S. used LCA to identify latent classes of high-risk behaviors and drug use ^31^. Based on the results, 4 latent classes were extracted as follows: low risk drinking / low prevalence drug use, lower intake drinking / moderate prevalence drug use, moderate risk drinking / moderate prevalence drug use, and high-risk drinking / high prevalence drug use. Another study in Canada used LCA and identified three latent classes of behavioral patterns as follows: 1) typical, 2) high risk, and 3) moderately healthy ^32^. Besides, a study in Iran evaluated high-risk behaviors among Iranian college students and identified three latent classes, namely 1) low risk, 2) smoking cigarette and hookah, and 3) high risk. Additionally, 3.7% of males and 0.4% of females were included in the high-risk class^20^. In the present study, LCA revealed that 79.7% and 2.5% of the sample belonged to low risk and very high risk classes, respectively, which is similar to other studies conducted in Iran^18, 20^.

Parental support and monitoring, as well as improved parent-child relationship, significantly decreased the probability of high-risk behaviors among the youth ,^9, 18, 33^. Our study results also suggested that higher parental support scores decreased the odds of membership in the second, third, fourth, and fifth classes in comparison to the first class.

The positive effects of religion and religious beliefs on reducing high-risk behaviors have been argued in the previous studies. As such, students with higher intrinsic religiosity and involvement in religious activities were less likely to engage in high-risk behaviors. In other words, vision played a significant role in getting engaged in healthy behaviors and avoiding high-risk behaviors ^3, 34^. The findings of the current study also suggested that higher religious beliefs scores decreased the odds of membership in the second, third, fourth, and fifth classes in comparison to the first class.

We showed that living alone increased the odds of all class memberships compared to the first class, with the highest odds ratio being related to the fifth class, supported earlier ^9, 35^.

The strengths of the present work were its relatively large sample size and high response rate, both of which increase the generalizability of the findings. One of the study limitations was using a self-administered questionnaire, which could lead to underestimation of the results. Additionally, this cross-sectional study was unable to explain the causal relationship between independent variables and high-risk behaviors. Future studies can focus on the longitudinal data about high-risk behaviors. Assessing the LCA and changing of the modeling in these studies with considering the related covariates seems ideally

## Conclusion

This study showed co-occurrence of high-risk behaviors by subgrouping a sample of university students into five classes. The results revealed that 2.5% of all students belonged to the very high-risk class. In addition, 7.7% of the students were in the high-risk class. These high rates emphasize the necessity to implement preventive interventions for this stratum of students. In addition, the results demonstrated that familial support and religiosity might serve as preventive factors in high-risk behaviors. Consequently, focusing on familial support and religious beliefs might be helpful in designing and executing effective preventative programs, which can be instrumental in the development of comprehensive health education programs with the goal of empowering individuals as well as the community.

## Acknowledgements

The present article was extracted from the M.Sc. thesis written by Sima Afrashteh and financially supported by Shiraz University of Medical Sciences (grant No.95-01-04-12407). Hereby, the authors would like to thank Ms. A. Keivanshekouh at the Research Improvement Center of Shiraz University of Medical Sciences for improving the use of English in the manuscript. They would also like to express their gratitude to all study participants.

## Conflict of interest statement

The author(s) declared no potential conflicts of interest with respect to the research, authorship, and/or publication of this article.

## Funding

The present article was financially supported by Shiraz University of Medical Sciences (Grant number 95-01-04-12407).

## Highlights

- Living alone (OR=3.11), higher score of religious beliefs (OR=0.36) and familial support (OR=0.33) and higher age (OR=0.79) associate with very high-risk class.
- Five latent classes were identified and 2.5% of the individuals are in the fifth class with a high probability of all indicators except physical violence
- The results suggested the protective effect of familial support and religiosity on high-risk behaviors.
